# Inter breath-hold reproducibility of temporal patterns of coronary artery blood flow

**DOI:** 10.1186/1532-429X-17-S1-M1

**Published:** 2015-02-03

**Authors:** Stephen Strain, Jennifer Keegan, Claire E Raphael, Robin Simpson, Malindie H Sugathapala, Sanjay K Prasad, David Firmin

**Affiliations:** 1 Royal Brompton Hospital, London, UK; 2 Radiological Physics, University of Freiburg, Freiburg, Germany; 3 NHLI, Imperial College, London, UK

## Background

While blood flow in the majority of arteries peaks in systole, the rhythmic squeezing of the coronary arteries and microcirculation as the heart beats results in left anterior descending artery (LAD) flow being diastolic-predominant while for the right coronary artery (RCA), there is approximately equal flow in systole and diastole. These temporal flow patterns can provide important information on disease state. The aim of this study is to assess the inter-breath hold reproducibility of parameters of coronary haemodynamics using a high temporal resolution spiral phase velocity mapping technique and to compare automatic and manual analyses.

## Methods

Two breath-hold coronary velocity maps were acquired in the proximal arteries of 10 LAD and 5 RCA (30 acquisitions in total): pixel size 1.4 x 1.4 x 8mm, true temporal resolution 19 ms, 1-1 water excitation (WE), 17 cardiac cycle breath-hold, retrospective ECG gating (reconstructed 50 phases). Extraction of coronary flow velocity-time curves was performed using a custom MATLAB program by two independent observers. Following Hough transformation, a circular coronary region of interest (ROI) was automatically defined on a through-plane segmented gradient echo scout image. This was copied to the spiral velocity maps and automatically shifted from frame to frame to track motion of the coronary through the cardiac cycle. For the LAD, a tracked arc-shaped ROI in nearby myocardium was used to correct for the through-plane motion of the vessel itself. For the RCA, the adjacent myocardium was too thin and a tracked ROI in the epicardial fat surrounding the vessel in a 1-1 fat-excitation (FE) breath-hold was used instead. Temporal flow profiles were extracted for each breath-hold acquisition and mean velocity (MV), peak systolic and diastolic velocities (PSV and PDV) and times to peak velocities (TPSV and TPDV) were determined. Bland Altman analysis and intraclass correlation coefficients (ICCs) were used to determine the inter breath-hold reproducibility of each parameter for each observer, and also to compare manual and automatic analyses. Results are quoted as mean +/- SD of signed differences between paired values.

## Results

**Figure 1:**
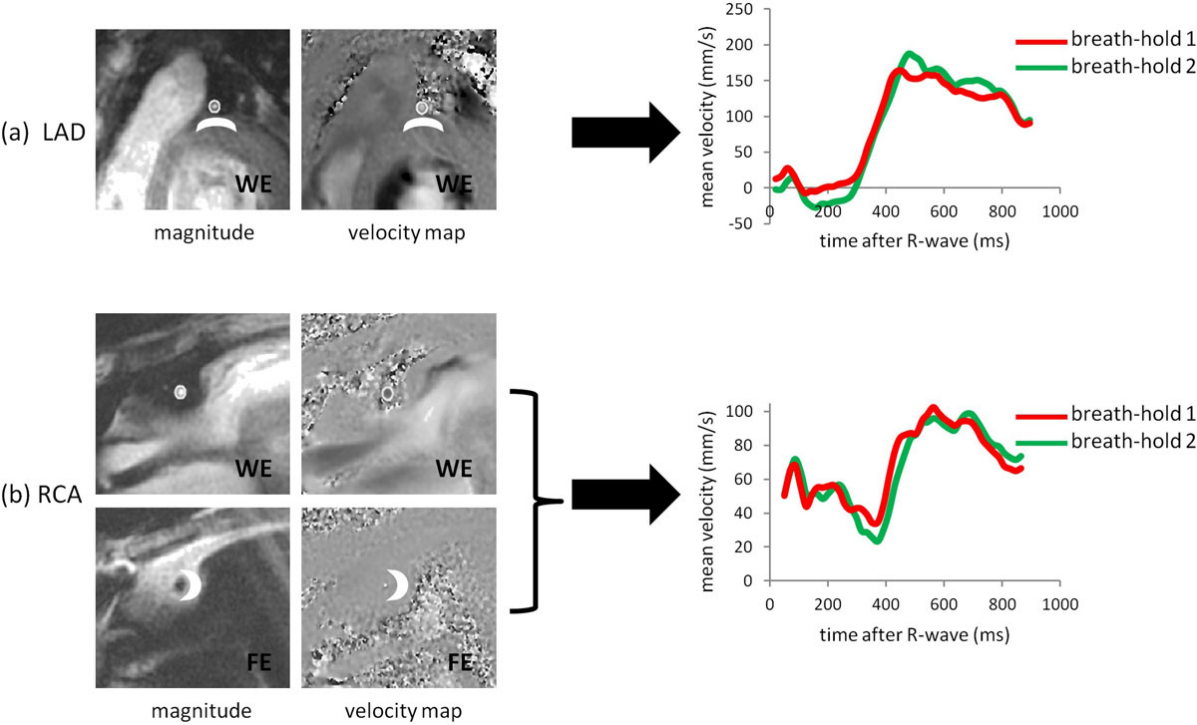
(a) Zoomed early diastolic water-excitation (WE) magnitude image and velocity map in an example left anterior descending (LAD) artery together with representative temporal flow profiles from repeated acquisitions. (b) Zoomed early diastolic WE and fat-excitation (FE) magnitude images and velocity maps in an example right coronary artery (RCA) together with representative temporal flow profiles from repeated acquisitions. In each image, coronary and through-plane correction regions of interest are shown (open circles and solid crescent shapes respectively).

Example data are shown in Figure 1. Automatic analysis typically took < 5 min (including review of ROIs in all 50 cine frames) and quantitative parameters agreed well with those obtained from tedious manual processing (taking ~30min). Inter breath-hold reproducbility of all parameters (mean +/- SD of signed differences and ICCs) and comparisons with manual analyses for each observer are shown in Table 1.

**Table 1:** mean +/- SD of paired differences and intraclass correlation coefficients (ICCs) for mean velocity (MV), peak systolic velocity (PSV), peak diastolic velocity (PDV), time to peak systolic velocity (TPSV) and time to peak diastolic velocity (TPDV). Inter breath-hold data and a comparison of manual and automatic analysis are presented for each observer (obs 1 and obs 2). Flow differences between manual and automatic processing (* p < .001) were due to differences in defining the cross-sectional area of the artery.

	MANUAL VERSUS AUTOMATIC ANALYSIS				INTER BREATH-HOLD REPRODUCIBILITY			
	mean +/- SD differences		ICC		mean +/- SD differences		ICC	

	Obs 1	Obs 2	Obs 1	Obs 2	Obs 1	Obs 2	Obs 1	Obs 2

MV (mm/s)	-0.9 +/- 9.8	-4.6+/-9.7	.97	.98	-2.4 +/-6.9	1.7+/-7.4	.98	.98

flow (mm3/s)	-175+/-184*	-251+/-214*	.90	.86	-32.6+/- 80.8	14.1+/91.0	.99	.99

SPV (mm/s)	-1.7+/-17	1.5+/-12.2	.99	.99	1.7+/-15.8	-11.8+/-46.0	.99	.92

TSPV (ms)	5.5+/-19.4	2.8+/-10.8	.97	.99	-4.4+/-22.1	-2.7+/-20.1	.99	.97

DPV (mm/s)	-1.3+/-24.7	-4.3+/-30.0	.98	.96	-3.5+/-11.6	2.9+/-21.4	.99	.98

TDPV (ms)	-3.0+/-50.2	-12.4+/-65.0	.93	.89	-0.1+/49.3	-20+/-70.3	.93	.87

## Conclusions

The automatic analysis technique reliably generates through-plane corrected temporal flow profiles with minimal user interaction and results agree well with those from manual analyses. Inter breath-hold reproducibility of MV, PSV, PDV, TPSV and TPDV (both observers) is excellent. Temporal patterns of coronary blood flow can be automatically assessed with high temporal resolution spiral phase velocity mapping.

## Funding

N/A.

